# Acute effects of low-volume intermittent versus higher-volume continuous exercise on arterial stiffness in healthy young men

**DOI:** 10.1038/s41598-022-05800-z

**Published:** 2022-02-02

**Authors:** Zhixiong Zhou, Lindong Hou, Mengnan Cui, Laurent Mourot, Weili Zhu

**Affiliations:** 1 Cardiovascular Health Laboratory, Capital University of Physical Education and Sports, 11 Bei San Huan Xi Road, Beijing, 100191 China; 2 EA3920 Prognostic Factors and Regulatory Factors of Cardiac and Vascular Pathologies, Exercise Performance Health Innovation (EPHI) platform, University of Bourgogne Franche-Comté, Besançon, France; 3 Division for Physical Education, National Research Tomsk Polytechnic University, Lenin Ave, 30, Tomsk Oblast, Russia 634050

**Keywords:** Cardiovascular biology, Circulation, Physiology

## Abstract

To compare the acute effects of low-volume intermittent and higher-volume continuous exercise on arterial stiffness, 20 healthy men (22.4 ± 0.4 years) were randomized to non-exercise control (CON), high-volume Continuous Exercise (CE), lower-volume Intermittent exercise of Long bouts with Long interval (ILL), of Long bouts with Short interval (ILS), and of Short bouts with Short interval trial (ISS). Exercise intensity was 35% heart rate reserve. Arterial stiffness in Cardio-ankle vascular index (CAVI) was measured at baseline (BL), immediately (0 min) and 40 min after exercise. CAVI changes from BL in the same trial (⊿CAVI) were used for analysis. There was no significant ⊿CAVI change in CON. ⊿CAVI decreased significantly at 0 min in all exercise trials, and reverted to baseline at 40 min only in CE and ILL. At 40 min, ⊿CAVI in ILS and ISS remained significantly lower than that of CON and CE. When ILS and ISS were compared with CON at 40 min, only ⊿CAVI in ISS remained significantly lower than that of CON. Despite low volume, the effect of intermittent exercise on arterial stiffness could be either equal or superior to that of higher-volume continuous exercise.

## Introduction

Cardiovascular disease (CVD) is the leading cause of mortality world wide, and its modifiable risk factors include smoking, overweight and obesity, diabetes, hypertension, dyslipidemia, lack of physical activity, unhealthy die and so on. Exercise can improve these risk factors, and hence prevent CVD. Besides continuous exercise, Guidelines from American College of Sports Medicine also recommend intermittent exercise as an alternative to maintain health^1^. Previous studies showed that intermittent exercise could be as effective as continuous exercise in improving CVD risk factors, such as glucose control^2^, fat utilization^3^, blood pressures^4^ and endothelial function^5^.

Arterial stiffness is another important independent risk factor for CVD^6^, and exercise can exert beneficial effects on it and hence prevent future CVD. Many studies had examined the acute effects of exercise on arterial stiffness in humans^7–9^. Recently, we had demonstrated that when exercise volume was matched, acute intermittent exercise elicited greater arterial stiffness decrease than continuous exercise^10–12^. When the interval was elongated, the acute superior effects of intermittent exercise disappeared^11^, while increasing number of bouts would maintain the effects of intermittent exercise even with elongated interval^12^, indicating that both interval duration and bout number could influence the effects of intermittent exercise on arterial stiffness. This is consistent with the opinion that manipulation of the number and duration of bouts and amounts of recovery can be used to produce a particular type of stress^13^. However, the current guideline proposed that exercise bout should be 10 min or more, not specifying the optimal bout number and interval duration between bouts^1^.

To further demonstrate the superiority of intermittent exercise, we designed intermittent exercise protocols of low volume, and compared them to a continuous exercise of higher volume. We hypothesized that low-volume intermittent exercise could be enhanced to greater extent by increasing bout number and shortening of interval duration, resulting in superior effects on arterial stiffness to continuous exercise of higher volume.

## Methods

### Subjects

Twenty healthy young men aged 22.4 ± 0.4 years participated in the study (Table 1). Informed consent was obtained from participants before the study. All participants were healthy and nonsmokers. None reported any disease known to affect the cardiovascular system. Ethics committee of Capital University of Physical Education and Sports approved all procedures (Ethical approval number: ST803692), and the study was carried out according to the Declaration of Helsinki.

**Table 1:** Subject characteristics (n = 20).

	Value (Mean ± SE)
Age (years)	22.4 ± 0.4
Height (cm)	176.5 ± 1.1
Weight (kg)	71.2 ± 2.0
BMI (kg/m^2^)	22.8 ± 0.5
Rest heart rate (beats/min)	61.0 ± 0.8
Systolic BP (mmHg)	122.7 ± 0.8
Diastolic BP (mmHg)	73.7 ± 0.7
CAVI	6.4 ± 0.1

Values are means ± SE.

BMI body mass index, BP blood pressure.

All participants abstained from vigorous activity and alcohol or caffeine intake over the day before the trial. Participants went to the laboratory fasting in the morning and rest quietly for at least 30 min before baseline measurement. All the trials were conducted with room temperature ranging from 22 to 25 °C in Cardiovascular Health Laboratory of Capital University of Physical Education and Sports.

### Design

**Figure 1:**
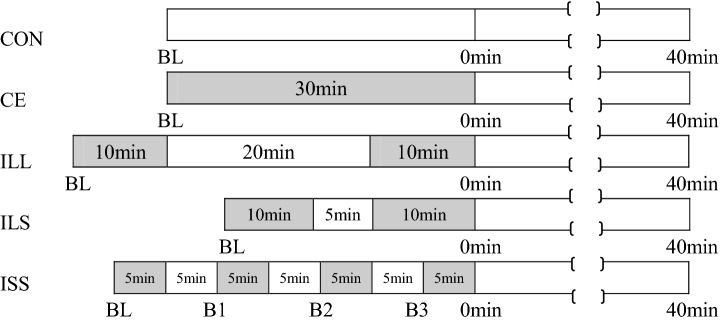
Study protocols for exercise, rest interval, recovery and measurements in five trials: non-exercise control trial (CON), high-volume continuous exercise trial (CE, one 30-min bout), low-volume intermittent exercise of long bouts with long interval (ILL, two 10-min bouts separated by 20-min interval), intermittent exercise of long bouts with short interval (ILS, two 10-min bouts separated by 5-min interval), and intermittent exercise of short bouts with short interval (ISS, four 5-min bouts separated by 5-min intervals). Time points of measurements were baseline (BL), immediately (0 min), and 40 min after exercise in CE, ILL, ILS and ISS trials.

Using a counterbalanced crossover self-control design, all subjects participated in five trials in random order, with each trial separated by 7 days. The trials were non-exercise control trial (CON), 30-min Continuous Exercise trial (CE), Intermittent exercise of Long bouts with Long interval (ILL, two 10-min bouts separated by 20-min interval), Intermittent exercise of Long bouts with Short interval (ILS, two 10-min bouts separated by 5-min interval), and Intermittent exercise of Short bouts with Short interval (ISS, four 5-min bouts separated by 5-min intervals), as seen in Fig. 1. These trials were performed between 7:00 AM and 11:00 AM following an overnight fast. Arterial stiffness was measured at baseline (BL), 0 min and 40 min post-exercise in the supine position. In CON trial, the subjects sat quietly except the measurements. In the exercise trail, following BL assessment, the subjects conducted the corresponding prescribed exercise on an ergometer (pedal frequency was 60 round per min).

In order to observe the arterial stiffness at the transition of interval to exercise bout in ISS, three more arterial stiffness measurements were made immediately before the second (B1), third (B2) and fourth 5-min bout, as indicated in ILL trial in Fig. 1.

### Exercise

Exercise was performed on an electrically braked bicycle ergometer (Aerobike 75XL, Combi, Tokyo, Japan). Intensity was set at 35% heart rate reserve, as used in our previous study^10^. The targeted heart rates were calculated using the Karvonen formula, which is: targeted heart rate = [220—age—resting heart rate] × 35% (exercise intensity 35%) + resting heart rate. During cycling, the heart rate of the participant was monitored by an ear photoelectric pulse sensor embedded in the ergometer, and the power rate was adjusted manually to keep the targeted heart rate of the participants during cycling.

### Measurement

Using a VaSera VS 1000 vascular screening system (Fukuda Denshi, Beijing, China), arterial stiffness was evaluated in Cardio-ankle vascular index (CAVI), an index of systemic arterial stiffness that reflects the condition of the aorta, femoral artery, and tibial artery. With the subjects in the supine position, electrocardiogram electrodes were placed on wrists, a microphone for monitoring heart sounds (phonocardiogram) was placed on the sternum, and four cuffs were wrapped around the upper arms and ankles. When the electrocardiogram was stable and the first and second heart sounds were detected in the phonocardiogram, the START button was pressed, and the values of right and left CAVI were obtained by the system completely independent of human operation ^10–12^. The average of the right and left CAVI was calculated, and its change from baseline in the same trial (⊿CAVI) used for later analysis.

Measurements of blood pressure and heart rate were made simultaneously by VaSera VS 1000 vascular screening system.

### Statistical analysis

All data are expressed as means ± SE if not mentioned otherwise. The response ⊿CAVI to exercise were analyzed by 4 separate two-factor repeated-measures ANOVAs in the form of 3 (group) × 3 (time), with Bonferroni post-test to determine the time point at which the significant difference between trials occurred. Mauchly's test of sphericity was performed to examine whether or not the assumption of sphericity was met. To observe the time course of ⊿CAVI dynamics in ISS trial, one-way ANOVA with repeated measures with Bonferroni post-tests was performed. The statistical significance level was set at *P* < 0.05. GraphPad Prism (version 8) was used for data analysis.

## Results

Table 1 shows the subject’s baseline characteristics including age, height, weight, body mass index and blood pressure. According to VaSera 1000 vascular screening system, the subject’s arteries were as old as their real ages.

Table 2 indicated that heart rate was significantly elevated at 0 min in CE, ILL, ILS and ISS trials (*P* < 0.01) compared to their baseline. Systolic blood pressure increased significantly at 0 min compared to its baseline in CE (*P* < 0.01).

**Table 2:** Effects of continuous and intermittent exercise on heart rate and blood pressure (*n* = 20).

	BL	0 min	40 min
**Heart rate, (beats/min)**			
CON	61 ± 2	57 ± 1	60 ± 2
CE	64 ± 2	111 ± 1*^†^	63 ± 3
ILL	61 ± 2	108 ± 1*^†^	58 ± 1
ILS	59 ± 2	108 ± 1*^†^	64 ± 5
ISS	61 ± 2	108 ± 1*^†^	59 ± 1
**SBP (mmHg)**			
CON	122 ± 2	121 ± 2	121 ± 2
CE	123 ± 1	128 ± 2*^†^	122 ± 2
ILL	124 ± 2	123 ± 3	124 ± 2
ILS	121 ± 2	125 ± 2	123 ± 2
ISS	124 ± 2	124 ± 4	121 ± 2
**DBP (mmHg)**			
CON	73 ± 1	74 ± 1	72 ± 2
CE	75 ± 2	73 ± 1	73 ± 2
ILL	73 ± 2	73 ± 1	77 ± 2
ILS	74 ± 2	72 ± 1	74 ± 1
ISS	74 ± 2	71 ± 1	74 ± 1

Values are mean ± SE.

BL baseline, CON control, CE continuous exercise trial, ILL intermittent exercise of long bouts with long interval, ILS intermittent exercise of long bouts with short interval, ISS intermittent exercise of short bouts with short interval, SBP systolic blood pressure, DBP diastolic blood pressure.

*P < 0.01 vs. BL; ^†^ *P* < 0.05 vs. CON.

**Figure 2:**
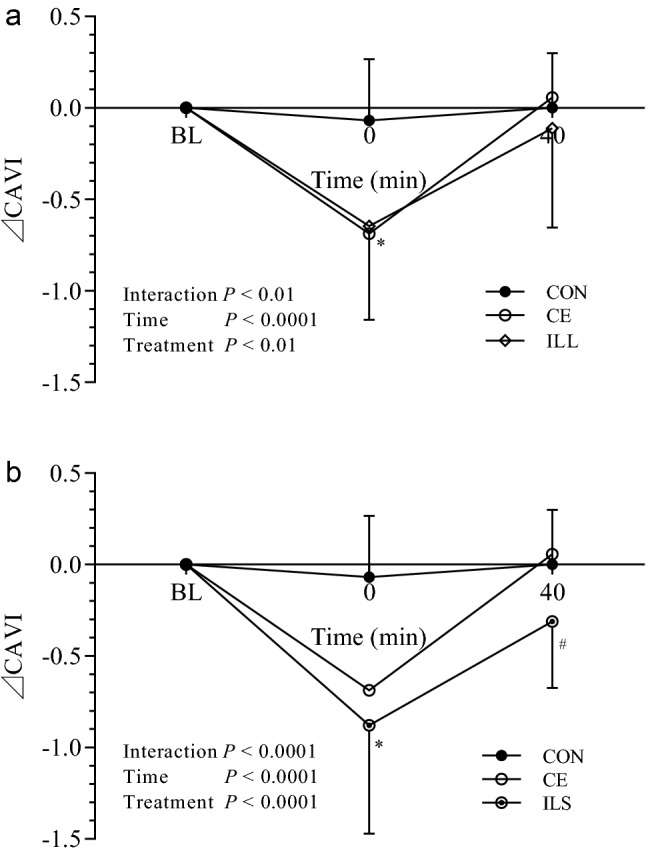
Time course of arterial stiffness response to exercise with and without interval of different duration. Mean (± SD) time-dependent ⊿CAVI changes in CON, CE and ILL trials (**a**) and CON, CE and ILS trials (**b**). Statistical analysis was performed using two-factor (treatment and time) repeated measures ANOVA with Bonferroni post-tests. Data are means ± SD, n = 20. *P < 0.0001, CE, ILL, ILS and ISS vs. CON at 0 min. ^#^*P* < 0.05, ILS vs. CON and CE at 40 min. CAVI Cardio-ankle vascular index, BL baseline, CON control, CE continuous exercise, ILL intermittent exercise of long bouts with long interval, ILS intermittent exercise of long bouts with short interval, ISS intermittent exercise of short bouts with short interval.

**Figure 3:**
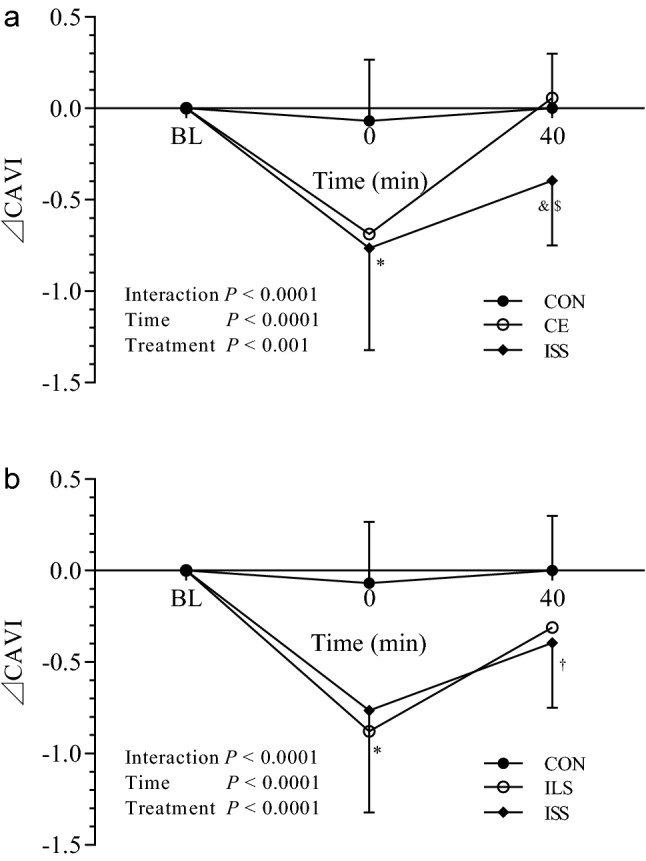
Time course of arterial stiffness response to exercise of different bouts with short interval. Mean (± SD) time-dependent ⊿CAVI changes in CON, CE, ISS trials (**a**), and CON, ILS and ISS trials (**b**). Statistical analysis was performed using two-factor (treatment and time) repeated measures ANOVA with Bonferroni post-tests. Data are means ± SD, n = 20. ^*^P < 0.0001, CE, ILS and ISS vs. CON at 0 min. ^&^*P* < 0.05, ISS vs. CON at 40 min. ^$^*P* < 0.01, ISS vs. CE at 40 min. ^†^*P* < 0.01, ISS vs. CON at 40 min.CAVI Cardio-ankle vascular index, BL baseline, CON control, CE continuous exercise, ILL intermittent exercise of long bouts with long interval, ILS intermittent exercise of long bouts with short interval, ISS intermittent exercise of short bouts with short interval.

The mean (± SD) changes of ⊿CAVI in the five trials were presented in Figs. 2 and 3. ⊿CAVI remained unaltered in CON trial (0.00 ± 0.00, − 0.07 ± 0.33 and 0.00 ± 0.30 at BL, 0 min and 40 min, respectively). ⊿CAVI changed with time in CE trial (0.00 ± 0.00, − 0.69 ± 0.51, 0.06 ± 0.72 at BL, 0 and 40 min, respectively), in ILL trial (0.00 ± 0.00, − 0.65 ± 0.51, − 0.11 ± 0.54 at BL, 0 and 40 min, respectively), in ILS trial (0.00 ± 0.00, − 0.88 ± 0.59, − 0.31 ± 0.36 at BL, 0 and 40 min, respectively), and in ISS trial (0.00 ± 0.00, − 0.76 ± 0.56, − 0.40 ± 0.36 at BL, 0 and 40 min, respectively).

As seen in Figs. 2 and 3, the treatment-by-time interaction reached significance (*P* < 0.01 in Fig. 2a and *P* < 0.0001 in Fig. 2b; *P* < 0.0001 in Fig. 3a and b), and this indicated that the time dependent ⊿CAVI changes were different between trials. The time main effects were also significant (*P* < 0.0001 in Figs. 2 and 3), indicating that ⊿CAVI changed significantly with time. Main effects of treatment reached significance (*P* < 0.01 in Fig. 2a,  *P* < 0.0001 i < 0.001 in Fig. 3a and *P* < 0.0001 in Fig. 3b).

Bonferroni post-test demonstrated that, at the time point of 0 min, ⊿CAVI in exercise trials was significantly lower than that in CON (*P < 0.0001, CE, ILL, ILS and ISS vs. CON, Figs. 2 and 3). At 40 min, ⊿CAVI in ILS was significantly lower than that in CON and CE trial (^#^*P* < 0.05, Fig. 2b), ⊿CAVI in ISS was significantly lower than that in CON (^&^*P* < 0.05, Fig. 3a) and CE trial (^$^*P* < 0.01, Fig. 3a), and ⊿CAVI in ISS, but not in ILS, was significantly lower than that in CON trial (^†^*P* < 0.05, Fig. 3b).

**Figure 4:**
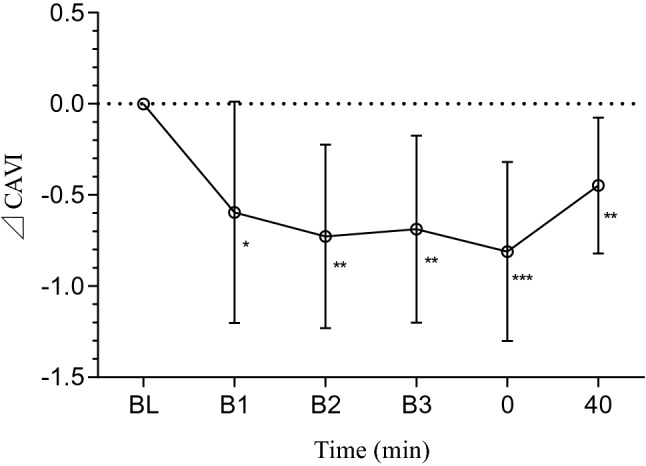
Mean (± SD) time-dependent ⊿CAVI changes undergoing ISS trial. CAVI was measured at baseline, immediately before the second (B1), third (B2) and fourth bout (B3) respectively, and immediately after exercise (0 min), and 40 min after exercise (Fig. 1). Statistical analysis was performed using one-way ANOVA with repeated measures with Bonferroni post-tests. Data are means ± SD, n = 13. ^*^P < 0.05 vs. BL; ^**^P < 0.01 vs. BL; ^***^P < 0.001 vs. BL. CAVI Cardio-ankle vascular index, BL baseline.

Figure 4 showed the CAVI changes relative to its baseline in ISS. ⊿CAVI in mean ± SD decreased significantly from 0.00 ± 0.00 at baseline (BL) to − 0.60 ± 0.61, − 0.73 ± 0.50, − 0.69 ± 0.51, − 0.81 ± 0.49, and − 0.45 ± 0.37 at B1, B2, B3, 0 min and 40 min, respectively.

## Discussion

The effects of intermittent and continuous exercise on arterial stiffness have been examined in both acute exercise^14^ and long term training^15,16^, and our previous studies have compared acute effects of intermittent and continuous exercise on a matched-volume basis^10–12^. To the best of our knowledge, this is the first study to compare the effects of low-volume intermittent exercise and higher-volume continuous exercise at the same low-intensity, on arterial stiffness in healthy young man. Our results showed that low-volume intermittent exercise could elicit at least equal, or even greater arterial stiffness improvement, compared to high-volume continuous exercise. The findings add to an emerging body of literature that short breaks with intermittent exercise contribute to decreased risk for CVD.

## Effects of interval and its duration of intermittent exercise on arterial stiffness

Long term training studies showed that intermittent exercise of higher intensity is more effective in decreasing arterial stiffness than moderate intensity continuous exercise^15,16^. Given the intensity-dependent relationship between exercise and cardiovascular responses^17^, it is difficult to distinguish whether the superior arterial stiffness improvement induced by intermittent exercise is due to its high-intensity nature or its intermittent nature. Hence, the significance of this study is that the exercise intensity of continuous and intermittent exercise is matched, so the effect of interval between bouts can be isolated.

A previous study demonstrated that long term accumulated brisk walking reduced arterial stiffness in sedentary, overweight individuals^18^. However, the interval between walking bouts was not mentioned. In our previous study, we showed that intermittent exercise in two 15-min bouts separated by 20 min produced greater arterial stiffness improvement than one 30-min bout of same intensity^10^. However, this was based on the matched total exercise volume of the two protocols. Using less exercise, the present study showed that two 10-min bouts of cycling separated by 20-min interval elicited arterial stiffness improvement to the same degree as one 30-min continuous cycling, which was of greater exercise volume. The inserted interval helped the two 10-min bouts to maintain the effects comparable to that of one 30-min bout (ILL in Figs. 1 and 2a), and the other advantage of two 10-min bouts is that people can save the time spent in cycling while preserving the benefit in arterial stiffness.

The superior effects of intermittent exercise might depend on the residual effect of the prior short exercise bout^19^, and we found that when the interval duration was elongated, the superior effects of intermittent exercise on arterial stiffness might disappear^11^. This inspired us to hypothesize that shortening the interval between bouts might enhance the effects of two 10-min bouts. Our results in this study showed that a protocol in two 10-min bouts with shorter interval (5 min) is superior to that of one 30-min continuous cycling (ILS in Figs. 1 and 2b). This is in agreement with the fact that a second bout of exercise elicited more pronounced change in immunoendocrine response when preceded by a short rest as opposed to a long rest after the first bout of exercise^20^. What is more, the total time of exercise and interval in ILS trial was reduced to 25 min, even shorter than the 30-min continuous exercise, so it represented the most time-saving protocol in this study.

## Effects of bout number on arterial stiffness with fixed short interval

Though the guidelines recommended exercise bout of 10–15 min in duration^1^, more information is needed on the benefits derived from very short (< or = 5 min) bouts of exercise^21^. Accordingly, several studies had adopted 5-min bouts in the protocol^22,23^, demonstrating the possibility and practice of very short bouts of exercise.

In this study, we designed intermittent exercise in the form of four 5-min bouts with 5-min intervals (ISS in Figs. 1 and 3a). Our results demonstrated its superior effects on arterial stiffness to one 30-min continuous bout, though not in agreement with the opinion that adults are likely to accrue similar health benefits from exercising in a single bout or accumulating activity from shorter bouts^24^. This might be associated with many factors, including the parameters used to evaluate the fitness, the exercise protocol adopted, and the participants involved.

We also compared the arterial stiffness improvement induced by four 5-min bouts with 5-min intervals to that of two 10-min bouts with the same 5-min interval (ISS and ILS in Figs. 1 and 3b). Though arterial stiffness decreased to similar extent immediately after exercise, arterial stiffness at 40 min in ILS showed no significant difference compared to CON at 40 min. In contrast, arterial stiffness after four 5-min bouts with 5-min intervals remained significant lower than that of CON at 40 min, indicating that intermittent exercise of four 5-min bouts could produce more arterial stiffness reduction than two 10-min bouts. The dynamic changes of arterial stiffness during exercise (Fig. 4) showed that CAVI decreased bit by bit after repeated 5-min bouts. This might be associated with the greater fluctuations in cardiac output during intermittent exercise, which modulated vascular shear stress, so the endothelium-derived nitric oxide (NO) was likely to be higher during intermittent exercise^14^. Zdrenghea, D., et al. demonstrated that prior exercise might enhance the production of NO of subsequent bout in humans^25^.

The present study has practical implication for the design of multiple short bouts completed within one hour. Less fatigue is a facilitator to exercise for people^26^, so the designs of more intervals and less total volume might increase exercise adherence. In a real-life setting, individuals were more inclined to exercise in multiple short-bouts per day, and enhanced exercise adherence^27^. So the results of this study might enhance the practice of intermittent exercise in regard of arterial stiffness improvement in humans.

There are several limitations in this study. First, this is an acute exercise intervention design, and the long term effects of intermittent exercise deserve attention. Second, result of the present study was based on healthy young men, and can not be simply extrapolated to other population such as patients and older people. Finally, the mechanism associated with the results is not explored.

## Summary

The main finding of the present study was that low-volume intermittent exercise with longer interval benefited arterial stiffness to the same extent as higher-volume continuous exercise in healthy young men. Shortening the interval of intermittent exercise resulted in greater arterial stiffness improvement, and the effects could be further enhanced by more fragmentation of the exercise.

The reduction of total exercise volume did not attenuate the superior effects of intermittent exercise on arterial stiffness compared to continuous exercise. The result of this study showed that both interval duration and bout number of intermittent exercise could play more important role in arterial stiffness regulation than total exercise volume.
